# Co-Ingestion of Whey Protein with a Carbohydrate-Rich Breakfast Does Not Affect Glycemia, Insulinemia or Subjective Appetite Following a Subsequent Meal in Healthy Males

**DOI:** 10.3390/nu8030116

**Published:** 2016-02-25

**Authors:** Dean M. Allerton, Matthew D. Campbell, Javier T. Gonzalez, Penny L. S. Rumbold, Daniel J. West, Emma J. Stevenson

**Affiliations:** 1 Faculty of Health and Life Sciences, Northumbria University, Newcastle upon Tyne NE1 8ST, UK; m.d.campbell@leedsbeckett.ac.uk (M.D.C.); j.t.gonzalez@bath.ac.uk (J.T.G.); penny.rumbold@northumbria.ac.uk (P.L.S.R.); daniel.west@newcastle.ac.uk (D.J.W.); emma.stevenson@newcastle.ac.uk (E.J.S.); 2 School of Sport, Carnegie Faculty, Leeds Beckett University, Leeds LS6 3QT, UK; 3 Department for Health, University of Bath, Bath BA2 7AY, UK; 4 Institute of Cellular Medicine, Medical School, Newcastle University, Newcastle upon Tyne NE2 4HH, UK

**Keywords:** whey protein, appetite, breakfast, glycemia, insulinemia

## Abstract

We aimed to assess postprandial metabolic and appetite responses to a mixed-macronutrient lunch following prior addition of whey protein to a carbohydrate-rich breakfast. Ten healthy males (age: 24 ± 1 years; body mass index (BMI): 24.5 ± 0.7 kg/m^2^) completed three trials in a non-isocaloric, crossover design. A carbohydrate-rich breakfast (93 g carbohydrate; 1799 kJ) was consumed with (CHO + WP) or without (CHO) 20 g whey protein isolate (373 kJ), or breakfast was omitted (NB). At 180 min, participants consumed a mixed-macronutrient lunch meal. Venous blood was sampled at 15 min intervals following each meal and every 30 min thereafter, while subjective appetite sensations were collected every 30 min throughout. Post-breakfast insulinemia was greater after CHO + WP (time-averaged area under the curve (AUC_0–180 min_): 193.1 ± 26.3 pmol/L), compared to CHO (154.7 ± 18.5 pmol/L) and NB (46.1 ± 8.0 pmol/L; *p* < 0.05), with no difference in post-breakfast (0–180 min) glycemia (CHO + WP, 3.8 ± 0.2 mmol/L; CHO, 4.2 ± 0.2 mmol/L; NB, 4.2 ± 0.1 mmol/L; *p* = 0.247). There were no post-lunch (0–180 min) effects of condition on glycemia (*p* = 0.492), insulinemia (*p* = 0.338) or subjective appetite (*p* > 0.05). Adding whey protein to a carbohydrate-rich breakfast enhanced the acute postprandial insulin response, without influencing metabolic or appetite responses following a subsequent mixed-macronutrient meal.

## 1. Introduction

There is a growing body of evidence implicating manipulation of meal macronutrient content, in particular protein ingestion, on markers of metabolic health (for review see [1]). The postprandial effects of dairy protein have been investigated extensively [2,3], and recent studies point to whey protein in particular as having potentially advantageous effects such as reduced postprandial glycemia [4,5] and lipemia [6], potentially mediated by an increased postprandial insulin response [7,8]. Recent studies also suggest that whey protein may induce potentially beneficial effects on appetite [9,10]. Despite the predominant use of fasting markers to determine disease risk in the clinical setting, the importance of the postprandial milieu including hyperglycemia [11] and hyperlipidemia [12] is increasingly being recognized as an important indicator of cardiovascular disease risk [13]. Thus, the use of sequential meal testing in individuals has a clear clinical utility, particularly with regard to triglyceride metabolism [14].

Ingestion of whey protein has been shown to promote insulin secretion to a greater extent than other proteins sources [15] in lean individuals, with a high concentration of branched-chain amino acids, particularly leucine, and their fast rate of appearance as likely integral mediators [16,17]. The subsequent synthesis and secretion of the incretin hormones glucose-dependent insulinotrophic polypeptide (GIP) and glucagon-like peptide-1 (GLP-1) in healthy subjects, due to the presence of amino acids and peptides derived from whey protein digestion [18], also likely play an important role in enhanced insulinemic response. Incretin hormones increase both fasting and postprandial insulin release [19]; their presence, in concert with increased plasma insulin concentrations, may also lead to appetite suppressive effects [20].

A number of studies have investigated the effects of whey protein when consumed prior to, or alongside, a meal on postprandial glycemia in normal-weight [15,21], overweight [9] or diabetic populations [22]. When whey protein (18.2 g) is ingested at the same time as a high glycemic index lunch meal, a 21% reduction in glucose area under the curve (AUC) has been identified in type 2 diabetes patients [5]. A dose-dependent reduction in glucose AUC has also been shown in healthy individuals [23]. Following consumption of whey protein 30 min prior to a meal, reductions in glycemia have also been shown in healthy participants [4] and type 2 diabetes patients [24]. However, the application of these data is reduced as the consumption of a preload approximately 30 min prior to a meal does not necessarily reflect conventional eating habits of the free-living individual [25].

Studies have also regularly prescribed large doses of whey protein, which may not be practical to consume and involve intake of relatively high amounts of additional energy; typically, doses of up to 55 g [22] have been employed. Furthermore, these studies typically involve short observation periods [26] and the observation of single meal responses [27]. The influence on subsequent feeding and metabolism is also important to understand, as the macronutrient composition of a breakfast meal has been demonstrated to influence the glycemic response following subsequent feeding [28] in healthy individuals. Thus, investigating the potential second meal effects of whey protein inclusion on glycemia, insulinemia, lipemia, and appetite may be important in identifying efficacious strategies to prevent potential declines in metabolic health, while providing further insights into the supplemental use of whey protein.

Therefore, the aim of this study was to determine the acute postprandial metabolic and appetite responses following a carbohydrate-rich breakfast, with and without co-ingestion of whey protein, in addition to responses following subsequent feeding.

## 2. Methods

### 2.1. Participants

Ten normal-weight male participants, free from metabolic disease, were recruited (mean ± SEM; age: 24 ± 1 years; mass: 79.7 ± 1.2 kg; stature: 1.81 ± 0.02 m; body mass index (BMI): 24.5 ± 0.7 kg/m^2^). Inclusion criteria included being recreationally active (>30 min of structured exercise, 5 times/week) and aged 18–40 years. Participants were not eligible if they were taking medication or had a history of metabolic disease, disordered eating or smoking. All participants provided written informed consent prior to inclusion, and all procedures were approved by the Faculty of Health and Life Sciences Research Ethics Committee, Northumbria University (Newcastle upon Tyne, United Kingdom) in accordance with the Declaration of Helsinki. This trial was registered at clinicaltrials.gov as NCT02414061.

### 2.2. Experimental Design

A crossover design was implemented with three experimental conditions consisting of a high-carbohydrate breakfast trial (CHO), a high-carbohydrate breakfast with added whey protein trial (CHO + WP) and a no breakfast trial (NB). Participants attended the laboratory on three separate occasions, separated by at least 72 h, and underwent all three conditions in random order. Prior to each visit, dietary intake was controlled through provision of a meal that participants were instructed to consume 12 h before arrival at the laboratory. This meal provided 1.0, 0.4 and 0.5 g/kg body mass of carbohydrate, fat and protein, respectively (3171 kJ). Participants were also instructed to avoid strenuous physical activity and to refrain from alcohol and caffeine consumption for 24 h prior to each visit.

**Figure 1:**
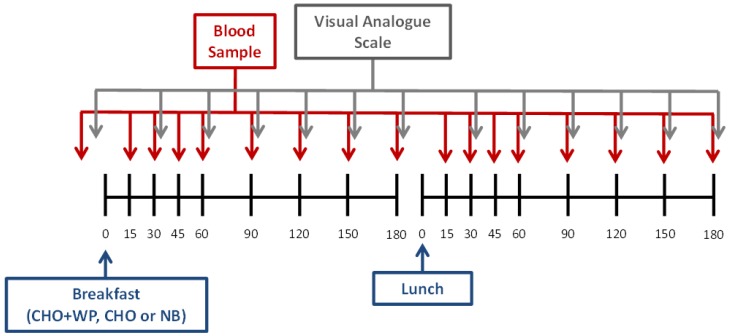
Schematic layout of experimental trials. CHO: high-carbohydrate breakfast trial; CHO + WP: high-carbohydrate breakfast with added whey protein trial; NB: no breakfast trial.

Upon arrival at ~0800 h, a cannula was inserted into an antecubital vein, and a baseline blood sample was collected (refer to 2.4 Blood Sampling and Analysis). Visual analogue scales (VAS) were also completed in order to assess appetite sensations at baseline (see 2.5 Subjective Ratings). Participants subsequently consumed one of two test breakfasts (CHO or CHO + WP) or ingested a reference bolus of water (NB) and remained in a rested state. Postprandial blood samples were collected at 15 min intervals for the first hour and every 30 min thereafter, while VAS were taken every 30 min throughout. After 180 min, participants were provided with the same standardized composite lunch meal under all conditions. Blood samples and VAS were subsequently collected at corresponding time points to the post-breakfast samples (Figure 1).

### 2.3. Test Meals

The energy content and macronutrient composition of the test meals provided during each trial are displayed in Table 1. The breakfast meal given under CHO and CHO + WP consisted of white wheat bread (95 g) and strawberry jam (48 g) with an orange juice beverage (200 mL), providing 1.2 g available carbohydrate/kg body mass (Table 1). In an effort to blind participants to the CHO and CHO + WP trials, 250 mL water was provided in an opaque bottle and flavored with 10 drops (approximately 0.5 mL) of energy-free vanilla flavoring under CHO. This process was repeated for the preparation of breakfast under CHO + WP, where an identical breakfast was provided albeit with the addition of 24 g whey protein isolate, which was dissolved in the flavored water. These beverages were therefore matched in terms of flavor; however, the difference in the consistency of the liquid due to the presence of the protein was not able to be accounted for. Participants may have been able to distinguish between these beverages; however, no reference was made to which trial was being conducted throughout. The whey protein powder (Arla Foods Ingredients Group, Viby, Denmark) had a protein content of 83%, providing 20 g protein per serving which has previously been identified as an efficacious dose for reducing glycemia [4]. The relative protein content of the CHO + WP breakfast was 0.4 g/kg body mass in comparison to 0.1 g/kg body mass under CHO. Water was also provided alongside breakfast, the volume of which was trial-dependent in order to standardize meal volumes across conditions. Under NB, a breakfast meal was not provided; however, an isovolumetric bolus (675 mL) of water was consumed by participants at the applicable time point. Further water was not administered during the postprandial period in order to maintain this standardization and control for any potential effects of meal volume on appetite suppression. A timer was started upon initiation of consumption in all trials, and participants were encouraged to consume the meal within 5 min.

An identical lunch meal was served at 180 min post-breakfast in all conditions. This consisted of a standard portion of pasta (125 g) that was cooked and added to a tomato-based sauce (170 g) along with cheddar cheese (40 g) and olive oil (15 mL) to provide 1.3, 0.4 and 0.4 g/kg body mass of carbohydrate, fat and protein, respectively. Water (350 mL) was also served alongside the lunch meal, and was withheld in the post-lunch period.

### 2.4. Blood Sampling and Analysis

At each collection point, a 10 mL sample of whole blood was drawn into a syringe from an intravenous cannula (Venflon, BD Becton Dickinson Ltd., Franklin Lakes, NJ, USA). A 20 µL capillary tube was filled with whole blood for immediate determination of glucose concentration by an automated analyzer (Biosen C_line, EKF Diagnostics, Cardiff, UK), while the remaining blood was transferred into an ethylenediaminetetraacetic acid (EDTA) tube (Vacutainer^®^, BD Becton Dickinson Ltd., Franklin Lakes, NJ, USA) and centrifuged (10 min, 1734× *g*, 4 °C). Plasma aliquots were stored at −80 °C for subsequent analysis. Following collection of each sample, the cannula was kept patent through infusion of saline solution. Plasma insulin concentration was analyzed using a commercially available enzyme-linked immunosorbent assay (ELISA) kit (IBL International, Hamburg, Germany), while triglyceride, non-esterified fatty acid (NEFA) and glycerol concentrations were determined using enzymatic colorimetric assays (Randox Laboratories, County Antrim, UK). Intra-assay coefficients of variation (CV) were <10% for all biochemical analyses.

### 2.5. Subjective Ratings

Subjective appetite ratings were recorded at baseline and at 30 min intervals following both breakfast and lunch meals using 100 mm paper-based VAS, which has been validated previously [29]. Scales were used to assess hunger, fullness, satisfaction and prospective food consumption, and were anchored with extreme statements of opposite meaning at each end, *i.e.*, “not at all full” and “totally full”.

### 2.6. Statistical Analysis

Subjective appetite scales were measured from the extreme left of the scale to the point where the participant had marked, in order to give a score in mm. All VAS were measured independently by two researchers with the mean score used in all subsequent analysis. Values for hunger, fullness, prospective consumption and satisfaction were used to calculate a combined appetite score by applying Equation (1) as used previously [30]:

(1) $$\text{combined appetite score}=\frac{\text{hunger}+\text{prospective consumption}+\left(100-\text{fullness}\right)+\left(100-\text{satisfaction}\right)}{4}$$

Time-averaged AUC data for the component aspects of subjective appetite were reported alongside this composite measure.

AUC values were calculated for subjective appetite and blood analyte data using the trapezoidal method for the post-breakfast (0–180 min) and post-lunch (180–360 min) periods [31], and these values were subsequently time-averaged. Statistical analysis was conducted using the Statistical Package for the Social Sciences (IBM SPSS; Version 21, Armonk, NY, USA). Baseline comparisons between trials and AUC for all variables were assessed using one-way repeated measures analysis of variance (ANOVA). The time point at 180 min post-breakfast was used as the baseline value for the post-lunch period. For combined appetite score, data were presented as change from baseline, with subsequent calculation of net incremental AUC (iAUC) [32]. Two-way repeated measures ANOVA (condition × time) was used to test for differences between plasma and appetite variables over time. Simple effects analysis was performed upon identification of a significant interaction effect, and the Bonferroni *post-hoc* test was used to correct the level of alpha for multiple comparisons. The level of statistical significance was set at *p* < 0.05 and data are presented as mean ± SEM.

## 3. Results

### 3.1. Post-Breakfast Responses

**Figure 2:**
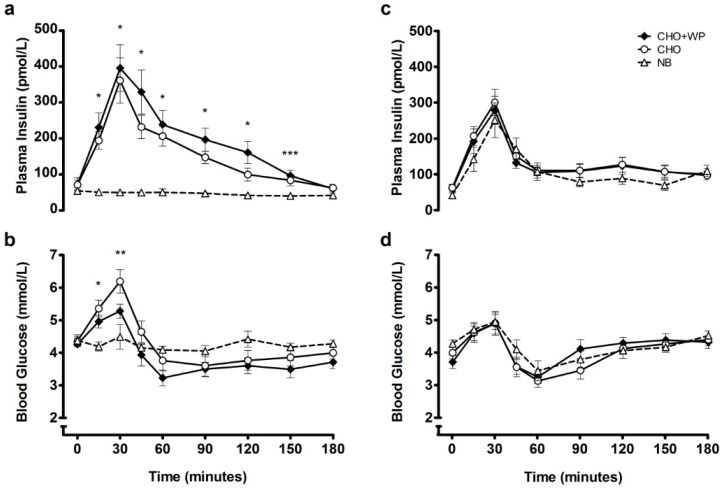
Mean ± SEM temporal changes in plasma insulin (**a**) and blood glucose (**b**) during the post-breakfast period, and plasma insulin (**c**) and blood glucose (**d**) during the post-lunch period. Significant differences (*p* < 0.05) between trials at individual time points are defined as follows: * NB *vs.* CHO + WP and CHO, ** NB *vs.* CHO, *** NB *vs.* CHO + WP. CHO: high-carbohydrate breakfast trial; CHO + WP: high-carbohydrate breakfast with added whey protein trial; NB: no breakfast trial.

**Figure 3:**
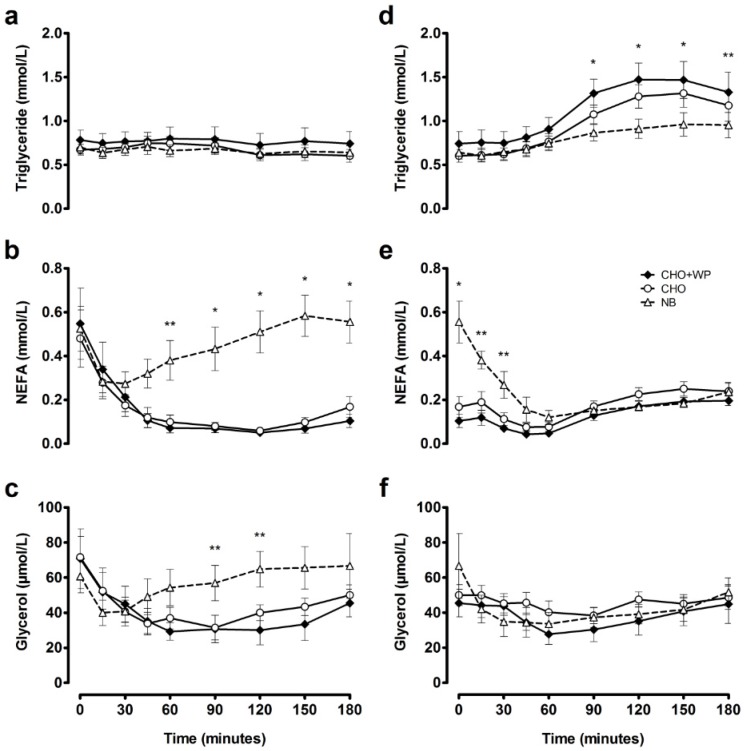
Mean ± SEM temporal changes in plasma triglyceride (**a**); NEFA (**b**) and glycerol (**c**) during the post-breakfast period, and plasma triglyceride (**d**); NEFA (**e**) and glycerol (**f**) during the post-lunch period. Significant differences (*p* < 0.05) between trials at individual time points are defined as follows: * NB *vs.* CHO + WP and CHO, ** NB *vs.* CHO + WP. CHO: high-carbohydrate breakfast trial; CHO + WP: high-carbohydrate breakfast with added whey protein trial; NB: no breakfast trial.

**Figure 4:**
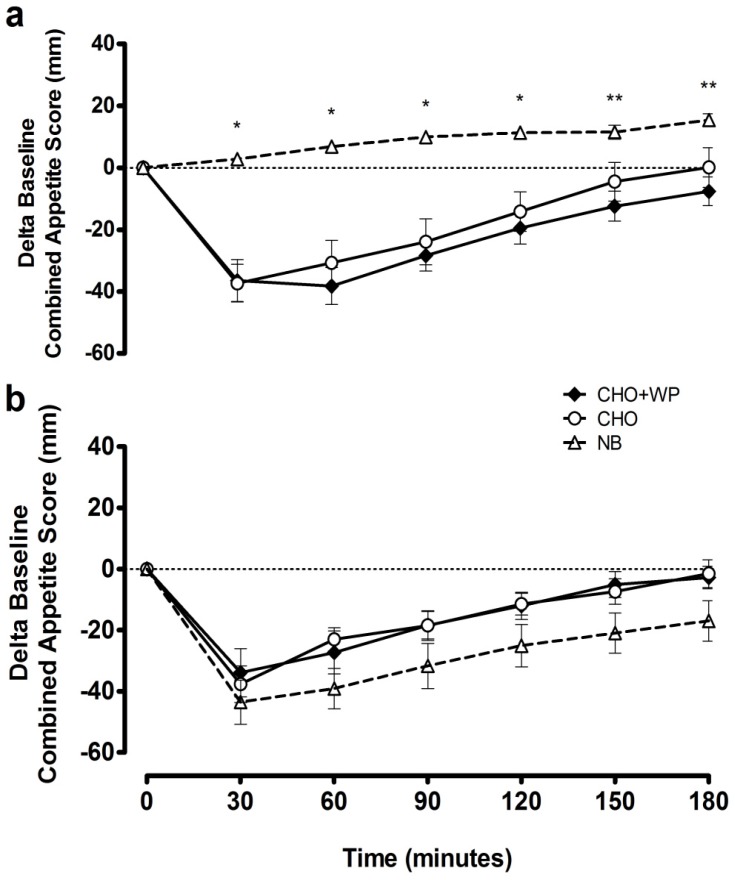
Mean ± SEM delta baseline changes in combined appetite score during the post-breakfast (**a**) and post-lunch (**b**) periods. Significant differences (*p* < 0.05) between trials at individual time points are defined as follows; * NB *vs.* CHO + WP and CHO, ** NB *vs.* CHO + WP. CHO: high-carbohydrate breakfast trial; CHO + WP: high-carbohydrate breakfast with added whey protein trial; NB: no breakfast trial.

Baseline measures for all variables were not different between trials (*p* > 0.05; Figure 2, Figure 3 and Figure 4). Post-breakfast plasma insulin concentrations are displayed in Figure 2a. There was a significant condition × time interaction (*p* < 0.001), condition effect (*p* < 0.001) and time effect (*p* < 0.001). Concentrations peaked 30 min after breakfast consumption under CHO + WP and CHO, with both similarly elevated above NB (*p* < 0.05) at 15–120 min post-breakfast, before returning to baseline levels. Insulinemia across this period was greater under CHO + WP than CHO (time-averaged AUC_0–180 min_: CHO + WP, 193.1 ± 26.3 pmol/L; CHO, 154.7 ± 18.5 pmol/L; *p* = 0.033), and was greater under both breakfast trials than NB (46.1 ± 8.0 pmol/L; *p* < 0.001; Table 2).

There was a significant condition × time interaction (*p* < 0.001) and significant time effect (*p* < 0.001) for blood glucose concentrations following breakfast consumption. Concentrations peaked similarly 30 min after both breakfast meals (Figure 2b), with no change observed under NB (*p* > 0.05). Blood glucose concentrations were not different between trials at 45–180 min post-breakfast (Figure 2b). Furthermore, there were no differences in glycemia between conditions across the postprandial period (Time-averaged AUC_0–180 min_: CHO + WP, 3.8 ± 0.2 mmol/L; CHO, 4.2 ± 0.2 mmol/L; NB, 4.2 ± 0.1 mmol/L; *p* = 0.247; Table 2).

Plasma concentrations of triglyceride, NEFA, and glycerol are presented in Figure 3. Triglyceride responses were not different between trials across the postprandial period following breakfast consumption (*p* > 0.05). NEFA concentrations were suppressed under CHO + WP and CHO in comparison to NB at 90–180 min post-breakfast (*p* < 0.05), while glycerol was significantly lower following CHO + WP than NB at 90–120 min post-breakfast (*p* < 0.05). NEFA time-averaged AUC_0–180 min_ was lower following both breakfast meals (CHO + WP, 0.13 ± 0.03 mmol/L; CHO, 0.13 ± 0.03 mmol/L) compared to NB (0.44 ± 0.08 mmol/L; *p* < 0.05; Table 2); however, triglyceride (CHO + WP, 0.77 ± 0.13 mmol/L; CHO, 0.67 ± 0.07 mmol/L; NB, 0.66 ± 0.07 mmol/L; *p* = 0.334) and glycerol (CHO + WP, 37 ± 7 µmol/L; CHO, 41 ± 5 µmol/L; NB, 57 ± 9 µmol/L; *p* = 0.092; Table 2) responses were not different between trials.

Temporal changes in subjective appetite following breakfast consumption are indicated by incremental time course responses for combined appetite scores (Figure 4a). There was a significant condition × time interaction (*p* < 0.001), condition effect (*p* < 0.001) and time effect (*p* < 0.001). Combined appetite scores were suppressed similarly under CHO + WP and CHO, with change from baseline under both remaining below NB from 30 to 120 min post-breakfast (*p* < 0.05). Time-averaged iAUC_0–180 min_ for combined appetite score was greater under NB in comparison to CHO + WP and CHO (*p* < 0.05; Table 3), with similar suppression observed under both breakfast trials (*p* > 0.05). This observation is replicated for feelings of hunger, while prospective consumption iAUC_0–180 min_ was greater under NB in comparison to CHO + WP only (*p* = 0.004; Table 3).

### 3.2. Post-Lunch Responses

Following lunch insulinemic and glycemic responses were not significantly different across all trials (Figure 2c–d). There was no effect of condition or time × condition interaction on plasma insulin or blood glucose concentrations (*p* > 0.05). Postprandial time-averaged AUC also indicated that insulinemia (CHO + WP, 130.7 ± 18.8 pmol/L; CHO, 136.9 ± 15.7 pmol/L; NB, 110.8 ± 18.6 pmol/L; *p* = 0.283) and glycemia (CHO + WP, 4.1 ± 0.1 mmol/L; CHO, 4.0 ± 0.1 mmol/L; NB, 4.1 ± 0.2 mmol/L; *p* = 0.509; Table 2) were not different throughout the 180-min period following consumption of a subsequent meal.

Plasma triglyceride concentrations were elevated above NB responses under CHO and CHO + WP at 90–150 min post-lunch (*p* < 0.05; Figure 3d). In contrast, no significantly different responses were observed between trials for NEFA from 45 to 180 min post-lunch (*p* > 0.05; Figure 3e), with no differences in plasma glycerol concentrations between trials throughout (*p* > 0.05; Figure 3f).

Deviations in subjective appetite responses between CHO + WP and CHO were not found following lunch (*p* > 0.05; Figure 4b). Indeed, time-averaged iAUC was not different between all conditions for the combined appetite score, in addition to the component measures of hunger, prospective consumption, and satisfaction (*p* > 0.05; Table 3). Post-lunch fullness iAUC was greater under NB than CHO + WP (*p* = 0.045), indicating more positive perception of this measure following prior breakfast omission (Table 3).

## 4. Discussion

The aim of this study was to investigate the effect of supplementing a carbohydrate breakfast with whey protein on metabolic and appetite responses acutely and following a subsequent meal. The study was designed to reflect the practical application in a free-living setting; the amount of whey, the timing of supplementation, and composition of meals were realistically achievable in the context of habitual eating habits. We show that adding 20 g of whey protein to breakfast increases the acute insulin response, without influencing glycemia, lipemia, or appetite sensations compared to an identical meal without additional whey in young healthy males. Here, for the first time, it is shown that adding 20 g of whey protein to breakfast does not alter glycemic, insulinemic, and appetite responses to a subsequent lunch meal, compared to breakfast without whey, or fasting, in young males free from metabolic disease.

Our observation of increased circulating insulin with whey protein supplementation reflects the effect observed in a number of studies [7,8,15]. Potential mechanisms may center on the amino acid profile of whey itself, and its consequent incretin hormone stimulatory properties [20]. While we detected a ~25% increase in post-breakfast (0–180 min) insulin AUC with the addition of whey to a carbohydrate breakfast, this did not augment a significant effect on postprandial blood glucose. This is contrary to the findings of previous studies, where various doses of whey protein have elicited positive effects on acute glycemia in both healthy [7,33] and diabetic [5,22] populations. This may partly be explained by insulin resistance being greater early in the morning following an overnight fast [34], suggesting that the insulinotrophic effect was not strong enough to offset this. Whey protein ingestion has also been shown to induce acute insulin resistance through impaired glucose disposal at whole body level and across the leg [35], albeit in obese post-menopausal female participants. While the mechanisms responsible for such an observation remain unclear, it is likely that any effects are counterbalanced by the promotion of insulin secretion by protein ingestion. While it is possible the dose of whey protein was insufficient to elicit a significant reduction in glycemia in healthy males, the efficacious dose to affect insulin and post-meal glycemic response is reported to be as low as 10 g in healthy individuals [4]. Following lunch, both glycemia and insulinemia were not affected by prior whey consumption, indicating the priming effect of elevated pre-lunch insulin on pancreatic beta cells may not have been substantial enough to alter post-lunch metabolism [36].

In the present study, whey protein supplementation did not affect subjective appetite. A number of studies have previously reported no effect of whey protein consumption on satiety and subsequent energy intake [37,38]. This is in contrast to other published observations, where consumption of whey has enhanced satiety through a variety of proposed mechanisms, including increases in peak amino acid, GLP-1, and cholecystokinin (CCK) concentrations in healthy females [9,39]. As energy intake was not measured here, it is difficult to infer any direct effect that these sensations may have on subsequent intake [40].

Cross-sectional studies have associated regular breakfast consumption with improved health outcomes such as lower BMI and improved glycemic control [41,42]; however, few randomized trials exist to support the causality of such associations, with recent studies suggesting no effect of breakfast consumption on weight loss [43,44]. In the current study, breakfast omission did not affect postprandial glucose or insulin concentrations following lunch. With regard to satiety, participants felt fuller (relative to baseline) following lunch after prior breakfast omission compared to after the whey protein-supplemented breakfast. This is surprising as breakfast omission has previously been shown to negatively affect satiety responses to foods later in the day, with [45] or without [46] subsequent increases in energy intake. The fact that participants were significantly less full at the lunch period baseline (180 min post-breakfast) after breakfast omission meant that, although the change in fullness from baseline was greater, this did not represent an absolute feeling of greater fullness. Breakfast omission did not significantly affect any of the other subjective appetite indices in the post-lunch period; similar circulating concentrations of insulin, which is considered a satiety hormone [47], reflected this. Further research is required to establish whether participants would overcompensate in terms of energy intake at a following meal regardless of subjective appetite ratings.

Measures of postprandial lipemia were similar between both breakfast trials throughout; however, the omission of breakfast appeared to impact on post-meal lipid metabolism. NEFA was significantly higher following breakfast omission, an effect that is likely mediated by the antilipolytic action of insulin released in response to breakfast [48], and returned to similar levels to both breakfast trials 45 min after lunch. Elevated NEFA levels have previously been linked to metabolic dysregulation and cardiovascular disease risk [49]; however, conversely, it has also been argued that they are not necessarily associated with insulin resistance [50]. Plasma triglyceride concentrations were significantly lower 90–150 min post-lunch following breakfast omission. It may have been concluded that such a finding is related to the appearance of triglyceride in the plasma following prior breakfast consumption [50]; however, the very low fat content of the breakfast (2 g) would suggest that the handling of dietary fat from lunch (33 g) was affected by breakfast omission. As insulin concentrations were not significantly different across conditions post-lunch, this suggests the reduction in plasma triglyceride concentration was not mediated by insulin-stimulated lipoprotein lipase activity. This reduction may have implications when considering the current swathe of public opinion suggesting that breakfast is the most important meal of the day. Our findings suggest that skipping breakfast on a single occasion may not be detrimental to the response to foods consumed later that day, and may provide beneficial effects on plasma triglyceride profile and, as mentioned above, subjective sensations of fullness. Whether subsequent energy intake would be influenced remains to be established; however, recent data indicates that the frequency, timing, and composition of meals consumed later in the day are not different during six weeks of breakfast omission compared to regular breakfast consumption [43]. Metabolic responses in the longer term are still unclear, however, with such behavior potentially leading to lower essential nutrient intakes over time.

The primary findings of the current study are contrary to the body of evidence that suggests whey protein can provide acute advantages in postprandial handling of meals. There is a clear need for studies investigating the effects of whey protein consumption at multiple meals so that findings can be applied to a real world setting. Research has focused on the effects of prior meal composition on responses to a subsequent meal, often termed the “second meal effect” [46,51,52]. In the present study, glycemia was not significantly different across trials following lunch. Chronic supplementation may be more efficacious when the aim is to positively affect the postprandial milieu throughout the day, particularly as individuals spend the majority of the day in the postprandial state [13]. Further research is also required to fully ascertain whether whey supplementation confers beneficial effects on metabolic responses. Acutely, the promotion of greater insulin secretion following whey protein consumption may have a glucose lowering effect [7,8,15]. Chronically, however, prolonged hyperinsulinemia in normoglycemic conditions may decrease insulin sensitivity [53]. The acute increase in circulating insulin observed in the present study may therefore be of benefit to individuals with type 2 diabetes; however, the chronic effects in healthy, insulin sensitive individuals is less clear.

The breakfasts provided in the present study were not matched for energy content, and this may limit the validity of direct comparisons between each meal. This was a consequence, however, of the effort to ensure that this experimental manipulation had practical application, in an effort to mimic the effect of “adding” a supplement to a meal, rather than replacing or substituting different components of a meal.

## 5. Conclusions

In summary, we implemented an ecologically valid protocol to assess the effect of supplementing a carbohydrate-rich breakfast with whey protein on metabolic and appetite responses, acutely and following a subsequent meal. Whey protein addition to breakfast increased the insulinemic response to that meal, without influencing metabolic or appetite responses to a subsequent mixed-macronutrient lunch. Breakfast omission induced glycemic, insulinemic, and combined appetite responses to a subsequent meal that, while maintaining lower circulating triglyceride, did not differ from breakfast consumption.

## Figures and Tables

**Table 1:** Energy and macronutrient composition of breakfast and lunch test meals.

		Energy kJ (kcal)	Carbohydrate g (E%)	Fat g (E%)	Protein g (E%)
	CHO	1799 (430)	93 (87)	2 (4)	9 (8)
Breakfast	CHO + WP	2172 (519)	95 (74)	2 (3)	29 (22)
	NB	0 (0)	0 (0)	0 (0)	0 (0)
Lunch		3446 (824)	104 (50)	33 (36)	29 (14)

E%: percentage of total meal energy; CHO: high-carbohydrate breakfast trial; CHO + WP: high-carbohydrate breakfast with added whey protein trial; NB: no breakfast trial.

**Table 2:** Time-averaged areas under the curve for blood and plasma variables during post-breakfast and post-lunch postprandial periods.

		CHO + WP ^1,2^			CHO			NB		
Glucose (mmol/L)										
Post-breakfast	AUC	3.8	±	0.2	4.2	±	0.2	4.2	±	0.1
	iAUC	−0.4	±	0.1	−0.2	±	0.1	−0.2	±	0.1
Post-lunch	AUC	4.1	±	0.1	4.0	±	0.1	4.1	±	0.2
	iAUC	0.4	±	0.2	−0.0	±	0.1	−0.1	±	0.2
Insulin (pmol/L)										
Post-breakfast	AUC	193.1	±	26.3 ^a^	154.7	±	18.5 ^b^	46.1	±	8.0 ^c^
	iAUC	120.2	±	15.3 ^a^	83.5	±	8.6 ^b^	−8.2	±	2.8 ^c^
Post-lunch	AUC	130.7	±	18.8	136.9	±	15.7	110.8	±	18.6
	iAUC	70.8	±	11.1	74.2	±	9.4	68.9	±	13.5
Triglyceride (mmol/L)										
Post-breakfast	AUC	0.77	±	0.13	0.67	±	0.07	0.66	±	0.07
	iAUC	−0.02	±	0.03	0.00	±	0.02	−0.04	±	0.02
Post-lunch	AUC	1.16	±	0.16 ^a^	0.99	±	0.11 ^a^	0.82	±	0.10 ^b^
	iAUC	0.42	±	0.05 ^a^	0.39	±	0.04 ^a^	0.18	±	0.05 ^b^
NEFA (mmol/L)										
Post-breakfast	AUC	0.13	±	0.03 ^a^	0.13	±	0.03 ^a^	0.44	±	0.08 ^b^
	iAUC	−0.42	±	0.13	−0.35	±	0.11	−0.08	±	0.05
Post-lunch	AUC	0.13	±	0.02 ^a^	0.18	±	0.02	0.21	±	0.02 ^b^
	iAUC	0.02	±	0.02 ^a^	0.01	±	0.03 ^a^	−0.35	±	0.08 ^b^
Glycerol (µmol/L)										
Post-breakfast	AUC	37	±	7	41	±	5	57	±	9
	iAUC	−34	±	15	−30	±	8	−4	±	4
Post-lunch	AUC	37	±	6	45	±	3	40	±	7
	iAUC	−9	±	4	−5	±	6	−26	±	13

^1^ Values presented as mean ± SEM; ^2^ Labeled values with different lowercase letters on the same row are significantly different between trials, *p* < 0.05. CHO: high-carbohydrate breakfast trial; CHO + WP: high-carbohydrate breakfast with added whey protein trial; iAUC: net incremental area under the curve; NB: no breakfast trial.

**Table 3:** Time-averaged areas under the curve for subjective appetite variables during post-breakfast and post-lunch postprandial periods.

		CHO + WP ^1,2^			CHO			NB		
Combined appetite score (mm)										
Post-breakfast	AUC	53.0	±	3.9 ^a^	55.4	±	4.6 ^a^	82.1	±	2.5 ^b^
	iAUC	−23.1	±	4.5 ^a^	−18.4	±	5.7 ^a^	8.3	±	0.8 ^b^
Post-lunch	AUC	52.2	±	4.8	57.4	±	5.2	61.0	±	5.7
	iAUC	−16.4	±	4.4	−16.5	±	3.5	−28.2	±	5.9
Hunger (mm)										
Post-breakfast	AUC	48.7	±	3.8 ^a^	52.5	±	4.3 ^a^	74.5	±	3.2 ^b^
	iAUC	−24.1	±	4.4 ^a^	−16.8	±	5.7 ^a^	9.8	±	2.1 ^b^
Post-lunch	AUC	48.7	±	4.7	54.8	±	5.0	57.3	±	5.4
	iAUC	−16.4	±	4.6	−17.0	±	3.8	−26.7	±	5.7
Fullness (mm)										
Post-breakfast	AUC	47.6	±	4.0 ^a^	46.1	±	4.6 ^a^	15.9	±	2.4 ^b^
	iAUC	25.9	±	4.7 ^a^	23.1	±	5.1 ^a^	−8.9	±	1.6 ^b^
Post-lunch	AUC	49.2	±	4.5	43.9	±	5.5	39.2	±	5.7
	iAUC	17.3	±	4.8 ^a^	17.8	±	3.4	30.8	±	6.2 ^b^
Prospective Consumption (mm)										
Post-breakfast	AUC	58.1	±	5.0 ^a^	60.4	±	6.0 ^a^	85.0	±	2.4 ^b^
	iAUC	−19.4	±	5.0 ^a^	−13.1	±	7.3	6.8	±	1.2 ^b^
Post-lunch	AUC	56.8	±	6.3	62.0	±	5.7	65.4	±	6.4
	iAUC	−15.6	±	4.6	−14.3	±	3.9	−24.0	±	6.4
Satisfaction (mm)										
Post-breakfast	AUC	47.1	±	3.5 ^a^	45.4	±	4.3 ^a^	15.4	±	2.5 ^b^
	iAUC	23.1	±	5.0 ^a^	20.7	±	5.1 ^a^	−7.8	±	1.7 ^b^
Post-lunch	AUC	47.6	±	4.1	43.2	±	5.1	39.7	±	5.5
	iAUC	16.1	±	4.8	16.8	±	3.5	31.1	±	5.7

^1^: Values presented as mean ± SEM; ^2^: Labeled values with different lowercase letters on the same row are significantly different between trials, *p* < 0.05. CHO: high-carbohydrate breakfast trial; CHO + WP: high-carbohydrate breakfast with added whey protein trial; iAUC: net incremental area under the curve; NB: no breakfast trial.
